# Age- and sex-related trends in body composition among Beijing adults aged 20–60 years: a cross-sectional study

**DOI:** 10.1186/s12889-023-16459-0

**Published:** 2023-08-10

**Authors:** Zhou Huayi, Xie Gang, Luo Laiyuan, He Hui

**Affiliations:** 1 Sport Human Science College, Beijing Sport University, Beijing, China; 2 National Physical Fitness Monitoring and Research Center of Wuxi Institute of Sports Science, Wuxi, China; 3 China Institute of Sport and Health Science, Beijing Sport University, Beijing, China; 4 China Institute of Sport and Health Science, Key Laboratory of Sports and Physical Fitness, Ministry of Education, Beijing Sport University, Beijing, China

**Keywords:** Age, Sex, Body composition, Obesity, Beijing adults

## Abstract

**Background:**

Obesity is the most serious global epidemic and body composition is the main indicator to evaluate obesity. This study aimed to investigate the changing trends of body composition by age and gender in Beijing adults aged 20–60 years and explore the distribution of obesity rates in different age groups of both sexes under different evaluation criteria.

**Methods:**

A total of 24,948 adults aged 20–60 years in Beijing, including 10,225 males and 14,192 females, were included, divided into four age groups (20–29, 30–39, 40–49, and ≥ 50 years) with each decade of age as an age group. Body composition indicators (BMI, fat mass, BF%, muscle mass, visceral fat area, and WHR) were measured in all subjects.

**Results:**

BMI and total fat mass peaked in males aged 40–49 years (BMI = 25.75 kg/m^2^, total fat mass = 17.70 kg). Female BMI, fat mass and BF% all increased significantly with age (*p* < 0.01). Total muscle peaked in males aged 30–39 years and decreased significantly thereafter (*p* < 0.0001). Visceral fat area and WHR increased significantly with age in both sexes (*p* < 0.0001). Age was significantly positively correlated with BMI, BF%, fat mass, WHR, and visceral fat area in both sexes (*p* < 0.0001), and age was negatively correlated with muscle mass in males (standard *β* =  − 0.14, *p* < 0.0001) while positive in female (standard *β* = 0.05, *p* < 0.0001). Under the BMI criterion, the obesity rate peaked at 27.33% in males at the age of 20–29 years. Under the BF% criterion, the obesity rate peaked at 17.41% in males at the age of 30–39 years, and increased in females with age. The central obesity rate of both sexes increased with age under the criteria of WHR and visceral fat area.

**Conclusion:**

The results of this study reveal that age- and sex-related patterns of body composition and obesity change among Beijing adults aged 20–60 years may differ across age groups and that such patterns of change should be considered when developing public health strategies.

**Supplementary Information:**

The online version contains supplementary material available at 10.1186/s12889-023-16459-0.

## Introduction

Obesity is the most serious global epidemic and can cause numerous adverse health consequences, including hypertension, diabetes, cardiovascular disease, and different types of cancer [1]. More than 2 billion people worldwide have become overweight or obese in the last decades, and 62% of these obese people live in developing countries [2]. Since the 1990s, the prevalence of overweight and obesity in China has steadily increased, and today, China ranks first in the world in terms of the overweight and obese population [3]. A recent public health study about obesity in China projected that by 2030, the overweight (24.0 kg/m^2^ ≤ BMI < 28.0 kg/m^2^) and obesity rates (BMI ≥ 28.0 kg/m^2^) among Chinese adults will reach 65.3% and 31.8%, respectively, and the medical costs of overweight and obesity will reach 418 billion yuan (approximately $61 billion), accounting for about 22% of the total medical costs, which will have a serious negative impact on the Chinese healthcare system and population health [4]. Effective and accurate measurement of obesity can not only reflect the nutritional status and health condition of the whole body but also provide valuable information for the diagnosis and treatment of various diseases [5].

The body composition ratio is an important indicator of overweight and obesity. Currently, the common techniques used to detect obesity include the underwater weighing method, air displacement plethysmography, computed tomography (CT), dual-energy X-ray absorptiometry (DXA), bioimpedance (BIA), ultrasound, etc. Among them, the BIA method has the advantages of simplicity of operation and accessibility and is mainly used for assessing body composition in the global clinical setting [6]. Body composition indicators measured by the BIA method include body mass index (BMI), fat mass, body fat percentage (BF%), skeletal muscle mass, waist-to-hip ratio (WHR), and other relevant indicators [7]. BMI is the most widely used indicator for evaluating overall obesity in clinical and population health research, and BMI has the advantage of being simple to measure and independent of height in assessing obesity [8], but a growing number of studies have shown that BMI does not distinguish between fat mass and muscle mass and is likely to incorrectly classify some individuals as healthy weight (i.e. those with increased fat mass and decreased skeletal muscle mass) and some as overweight (i.e. those with increased skeletal muscle mass and relatively low fat mass) [9]. BF% is also a common indicator for evaluating overall obesity, and some studies have shown that BF% is effective in identifying normal weight and overweight people at high cardiometabolic risk compared to BMI [10]. Waist circumference and visceral fat area are common indicators for evaluating abdominal obesity, and numerous studies have shown that abdominal obesity is a stronger predictor of associated health risks (e.g. cardiovascular disease, cancer, type 2 diabetes, dementia, and depression) than overall obesity [10, 11].

Many factors influence obesity, with age and gender being two of the most important factors. Kuk et al. found that characteristic changes in body composition with age, mainly in the form of decreased muscle mass, increased fat mass, and redistribution from the periphery to the abdomen [12]. Therefore, central obesity is more prevalent in older populations, and studies have found that the prevalence of central obesity in people over 40 years of age is approximately twice as high as in people aged 15–40 years [13]. Obesity is also influenced by region, diet, lifestyle, physical activity, and education. Beijing, the capital city, currently exhibits urban diets and socioeconomics that are characteristic of developed country consumption patterns [14]. While most studies on obesity in China to date have been conducted in urban and/or high-income settings, few have addressed the Beijing population alone. Studying the body composition of the Beijing population and the distribution characteristics of obesity by age and sex would also be beneficial in specifying public health strategies in other similar rapidly developing cities and would be important in preventing the development of obesity. Therefore, this study shows the trends of age and sex of human body composition in a large age range with a large-scale sample covering adults aged 20–60 years in Beijing, China. In addition, this study explores the characteristics of overall and central obesity changes among different ages and sexes under the evaluation criteria of BMI, BF%, WHR, and visceral fat area, providing important data for the distribution of obese adults in Beijing, China, and providing strong theoretical guidance for the detection and management of obesity-related chronic diseases.

## Methods

### Ethics committee statement

This study followed the principles of the Declaration of Helsinki of the World Medical Association and was approved by the Beijing Sport University Committee for a cross-sectional study. Informed written consent was obtained from all participants prior to their participation in this study.

### Subjects

Subject recruitment for this study was conducted in the main urban areas of Beijing (Chaoyang District, Dongcheng District, Xicheng District, Haidian District, Fengtai District, and Shijingshan District). Five streets were first randomly selected according to the street division of each urban area, and then the selected streets were used as the center for advertising, and those who were interested in participating in this study responded by email, telephone, and WeChat. A total of 25,263 adults responded to participate in this study, and all participants completed a questionnaire that included background information on socio-demographic characteristics, gynecological information, disease information, and physical activity levels. The study was restricted to adults aged 20–60 years and subjects with the following criteria were excluded based on questionnaire information: 1) having any neuromuscular, severe liver, heart, or kidney dysfunction cancer or other diseases capable of affecting body composition; 2) taking medications that may affect body composition, such as glucocorticoids, anticonvulsants, bisphosphonates, thyroxine, and vitamin D; 3) having implanted electronic devices such as pacemakers; 4) those under dietary supervision or regular exercise; 5) those with somatic deformities, such as skeletal deformities and walking disorders; and 6) pregnant or and lactating women. In the end, 24,417 subjects met the requirements of the experiment and completed all tests, of which 10,225 were men and 14,192 were women.A randomized whole-group sample of adults aged 20–60 years was drawn from the Dongcheng, Xicheng, Haidian, and Chaoyang districts of Beijing. A total of 24,948 adults participated in this study, of whom 10,225 were male and 14,192 were female.

### Measurements

Body composition was measured by bioelectrical impedance technology using a body composition analyser (JAWON-X-SCAN PLUS, Korea), and the testing environment was maintained at a suitable temperature (20–25 °C). The subjects should fast for 12 h before the test and test at 7–9 am, empty the urine and stool before the test, stand still for 5 min and then remove the items in the pockets and other belongings, and step on the electrode with bare feet in the shape of the foot-shaped electrode of the instrument. The subject gently held the handle electrode with the thumb and fourth finger all the way through, with the arm extended at an angle of 15 degrees to the torso. Throughout the test, the subjects were tested in an upright, stationary position. Test parameters included body weight, BF%, fat mass, muscle mass, muscle mass by segment, and visceral fat area.

### Height, waist, and hip measurements

To measure height, the subjects removed their shoes, rested their back against the height tester, straightened the back, and positioned the head, looking straight ahead, with arms hung naturally, legs together and straightened, and feet together. Then, the tester slowly moved the horizontal plate on the tester down to make contact with the tester’s head and then stopped. After the tester’s dashboard data did not fluctuate, the height data was recorded in centimetres and one decimal place was retained. The BMI was calculated from measured height and weight data: BMI (kg/m^2^) = W (kg)/H^2^ (m^2^). When measuring waist circumference, the tester remained relaxed and kept the back naturally straight. The left and right hands were interspersed, and the tester stood on the subject’s side, removed the clothes covering the subject’s waist, placed the soft ruler 1–2 cm from the upper edge of the iliac bone, and then circled along the abdominal skin to ensure no extrusion and deformation between the skin. The waist circumference was measured in centimetres in the subject’s normal breathing state. To measure the hip circumference, the soft ruler was placed around the hip projection for one week and the tightness of the soft ruler was controlled to ensure no extrusion deformation between the skin; the unit is centimetre. The WHR was calculated from measured waist circumference and hip circumference. The relevant personnel involved in the measurement were uniformly trained to ensure the standardization of the test and the reliability of the data.

### Obesity criteria

According to the BMI evaluation criteria for obesity recommended by the Chinese Health Care Commission [15], 18.5 kg/m^2^ ≤ BMI ≥ 24 kg/m^2^ is considered normal, and a BMI of > 24.0 kg/m^2^ and ≥ 28.0 kg/m^2^ is considered overweight and obese, respectively. According to the definition of BF% [16], BF% ≥ 28.0% in men and BF% ≥ 30.0% in women were used as the overall obesity evaluation criteria. WHR and visceral fat area were commonly used to evaluate central obesity; WHR ≥ 0.90 in men, WHR ≥ 0.85 in women, and visceral fat area ≥ 100 cm^2^ were usually used as the central obesity evaluation criteria [17, 18].

### Statistical analysis

All data were pre-processed using Excel 2010 (indicator merging, data error checking, etc.) and analysed using IBM SPSS Statistics 23 and StataSE 16 statistical software. The Kolmogorov–Smirnov and Levene tests were used to assess whether the variables conformed to normal and chi-square distribution, respectively, and the test results were used for descriptive statistics with median, interquartile range (IQR), and range of most values (minimum to maximum). Comparisons between different age groups were made using the K independent-sample nonparametric test, and the Kruskal–Worris method was used for two-way comparisons between groups and the Mann–Whitney U test for two-way comparisons between different genders. The least mean square (LMS) method was used to generate percentile curves (10th, 25th, 50th, 75th, 90th, and 95th percentiles) for these non-normally distributed data. The percentage of obesity was calculated by gender under different obesity criteria, and the quantile regression coefficients between age and body composition indicators were calculated for different genders. For all tests, *p* < 0.05 indicated a significant difference, and *p* < 0.01 indicated a highly significant difference.

## Results

### Participant characteristics

The general characteristics of the body composition of all subjects are listed in Table 1. BMI, BF%, fat mass, muscle mass, left and right upper limb fat, left and right lower limb fat, trunk fat, visceral fat area, and WHR were statistically different between males and females (*p* < 0.0001). Other body composition indicators were significantly higher in males than in females, except for BF% (*p* < 0.0001). The data in Table S1 shows that adults aged 20–60 have a variety of occupations and the percentage distribution of each occupational group is similar.

**Table 1:** Descriptive statistics for all observations of males and females

	Men, *n* = 10,225		Women, *n* = 14,192		*p* (sex)
	Median (IQR)	Range	Median (IQR)	Range	
Age (year)	37.00 (17.00)	20.00–60.00	37.00 (14.00)	20.00–60.00	
BMI (kg/m^2^)	22.90 (7.20)	15.80–49.90	22.80 (4.60)	13.60–46.00	< 0.0001
BF%	25.40 (4.60)	3.00–52.70	27.20 (7.50)	3.30–46.00	< 0.0001
Total fat (kg)	17.50 (8.40)	1.30–59.40	16.10 (7.60)	1.50–56.90	< 0.0001
Total lean mass(kg)	54.20 (8.10)	32.20–92.90	40.00 (5.10)	27.90–67.60	< 0.0001
Left arm muscle (kg)	3.71 (0.59)	0.71–14.09	2.63 (0.37)	0.35–53.51	< 0.0001
Right arm muscle (kg)	3.68 (0.60)	0.53–12.23	2.59 (0.39)	0.34–54.04	< 0.0001
Left leg muscle (kg)	10.02 (1.60)	6.20–40.81	7.58 (1.07)	5.10–99.24	< 0.0001
Right leg muscle (kg)	9.92 (1.57)	3.17–19.40	7.47 (1.03)	1.32–46.81	< 0.0001
Trunk muscle (kg)	26.84 (3.88)	13.09–45.13	19.68 (2.37)	6.08–622.78	< 0.0001
Visceral fat area (cm^2^)	99.00 (47.00)	20.00–367.00	43.00 (34.00)	20.00–229.00	< 0.0001
WHR	0.89 (0.09)	0.64–1.16	0.79 (0.09)	0.64–1.05	< 0.0001

All data are presented as median (interquartile range)

*BMI* body mass index, *BF%* percent body fat, *WHR* waist-to-hip ratio

### Body composition changes with age and gender

**Fig. 1:**
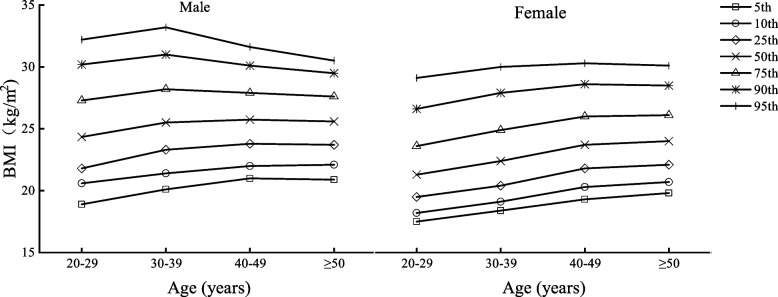
BMI percentile curves by age groups in the two genders

**Fig. 2:**
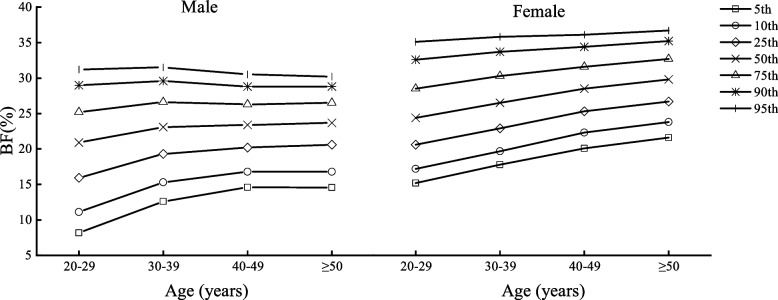
BF% percentile curves by age groups in the two genders

**Fig. 3:**
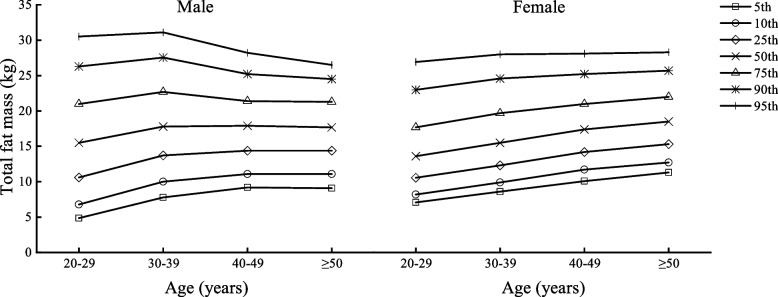
Total fat mass percentile curves by age groups in the two genders

**Fig. 4:**
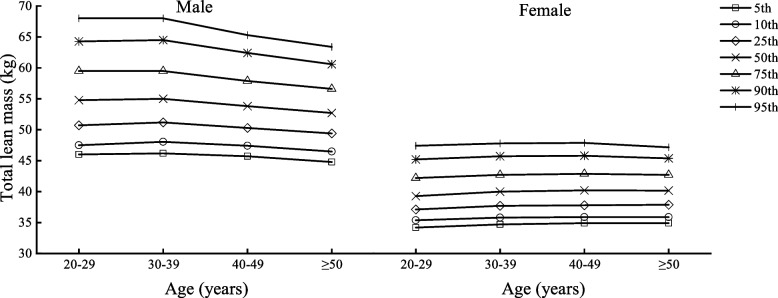
Total lean mass percentile curves by age groups in the two genders

**Fig. 5:**
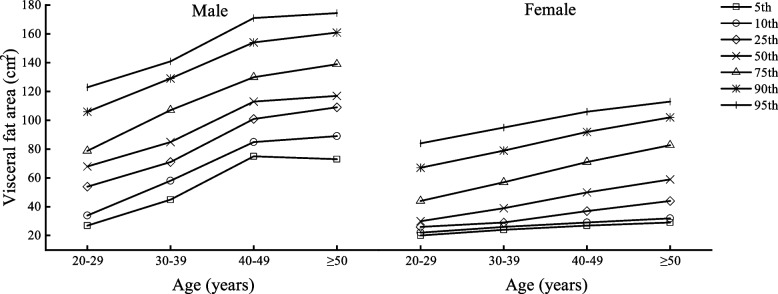
Visceral fat area percentile curves by age groups in the two genders

**Fig. 6:**
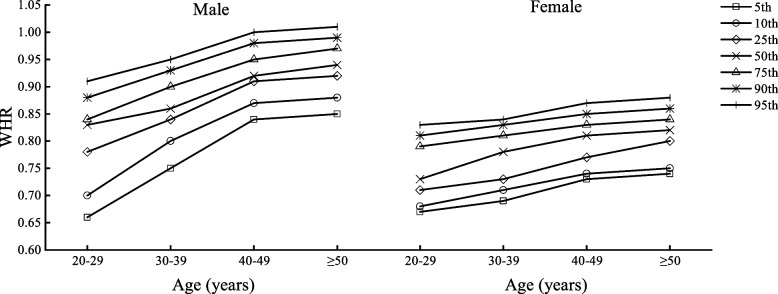
WHR percentile curves by age groups in the two genders

Table 2 shows the body composition characteristics of Beijing adults by age and sex, divided into four age groups (20–29, 30–39, 40–49, and ≥ 50 years) with one age group for each decade. Figures 1, 2, 3, 4, 5 and 6 display the age and gender curve distributions of the body composition indicators of the subjects. The data in Table 2 show that BMI, total muscle mass, muscle mass of body segments, visceral fat area, and WHR were significantly higher in males aged 20–60 years than in females (*p* < 0.0001), and BF% and total fat mass were significantly higher in females than in males (*p* < 0.0001). BMI, BF%, and total fat mass increased significantly (*p* < 0.05) in males aged 30–39, 40–49, and ≥ 50 years compared to 20–29 years. BMI and total fat mass peaked at 40–49 years (BMI = 25.75 kg/m^2^, total fat mass = 17.70 kg), with a slightly decreasing trend thereafter, whereas both BMI and BF% gradually increased with age in females. There was a significant increase in the 30–39-, 40–49-, and ≥ 50-age groups compared to the previous age groups (*p* < 0.01). Total lean mass and muscle mass in all body segments peaked in males at 30–39 years of age, and they were significantly smaller in each of the latter age groups than in the previous age groups (*p* < 0.0001). While muscle mass of all segments except trunk muscle mass was significantly higher in females aged 30–39, 40–49, and ≥ 50 years than in females aged 20–29 years (*p* < 0.0001), there was a significant increase in muscle mass of the left and right upper limbs in the 40–49-year and ≥ 50-year groups compared to the 30–39-year group (*p* < 0.05) and in muscle mass of the left and right lower limbs in the 40–49-year and ≥ 50-year groups compared to the 30–39-year group (*p* < 0.05). Visceral fat area and WHR increased significantly with age in both males and females and were significantly higher in each age group after 30–39 years compared with the preceding age groups (*p* < 0.0001). Notably, the greatest increase in visceral fat area with age was observed in the ≥ 50-year group for both sexes compared to the 20–29-year group, which approximately doubled. In conclusion, the distribution of body composition showed different characteristics by gender with age, and both fat and muscle mass decreased significantly with age in males after 40 years of age, while fat mass increased with age and muscle mass changed less in females, and visceral fat area and WHR increased significantly and substantially with age in both genders.

**Table 2:** Body composition changes with age and gender

	20–29 years	30–39 years	40–49 years	≥50 years
	*n* = 5253 (2296♂; 2957♀)	*n* = 8325 (3241♂; 5084♀)	*n* = 7054 (2598♂; 4459♀)	*n* = 3782 (2090♂; 1692♀)
	Median (IQR)	Median (IQR)	Median (IQR)	Median (IQR)
Age (year)	27.00 (3.00)	34.24 (4.00)	45.00 (5.00)	53.00 (4.00)
Male	22.50 (4.00)	34.00 (4.00)	45.00 (5.00)	54.00 (5.00)
Female	27.00 (3.00)	34.00 (5.00)	45.00 (5.00)	52.00 (3.00)
BMI (kg/m^2^)	22.50 (5.40)	23.70 (5.40)^a^	24.50 (4.60)^ab^	24.90 (4.10)^abc^
Male	24.35 (5.47)	25.50 (4.90)^a^	25.75 (4.10)^a^	25.60 (3.90)^a^
Female	21.30 (4.10)***	22.40 (4.50)^a^***	23.70 (4.20)^ab^***	24.00 (4.00)^abc^***
BF%	22.90 (8.60)	25.10 (7.50)^a^	26.60 (7.20)^ab^	26.30 (7.30)^ab^
Male	20.90 (9.27)	23.10 (7.30)^a^	23.40 (6.10)^a^	23.70 (5.90)^ab^
Female	24.40 (7.90)***	26.50 (7.40)^a^***	28.50 (6.30)^ab^***	29.80 (6.00)^abc^***
Total fat (kg)	14.30 (8.70)	16.30 (8.30)^a^	17.50 (6.90)^ab^	18.10 (6.80)^abc^
Male	15.50 (10.40)	17.80 (9.00)^a^	17.90 (7.02)^a^	17.70 (6.90)^a^
Female	13.60 (7.15)***	15.50 (7.40)^a^***	17.40 (6.80)^ab^***	18.50 (6.67)^abc^***
Total lean mass (kg)	44.40 (15.10)	44.50 (14.40)^a^	43.40 (12.50)^ab^	47.30 (12.70)^abc^
Male	54.80 (8.77)	55.00 (8.30)	53.80 (7.60)^ab^	52.70 (7.20)^abc^
Female	39.30 (5.10)***	40.00 (5.00)^a^***	40.20 (5.10)^a^***	40.15 (4.80)^a^***
Left arm muscle (kg)	2.94 (1.15)	2.96 (18.59)	2.90 (0.95)^ab^	3.22 (0.96)^abc^
Male	3.73 (0.62)	3.76 (0.61)	3.70 (0.56)^ab^	3.62 (0.53)^abc^
Female	2.56 (0.38)***	2.63 (0.36)^a^***	2.66 (0.37)^ab^***	2.67 (0.36)^ab^***
Right arm muscle (kg)	2.91 (1.15)	2.93 (1.09)^a^	2.85 (0.97)^b^	3.18 (0.98)^abc^
Male	3.71 (0.62)	3.73 (1.70)	3.68 (0.57)^b^	3.60 (0.55)^abc^
Female	2.53 (0.39)***	2.59 (0.38)^a^***	2.62 (0.39)^ab^***	2.63 (0.37)^ab^***
Left leg muscle (kg)	8.37 (2.64)	8.44 (2.50)^a^	8.29 (2.21)^b^	8.84 (2.23)^abc^
Male	10.12 (1.70)	10.16 (1.70)	9.98 (1.54)^ab^	9.77 (1.44)^abc^
Female	7.44 (1.02)***	7.58 (1.07)^a^***	7.65 (1.09)^ab^***	7.62 (1.07)^a^***
Right leg muscle(kg)	8.27 (1.84)	8.33 (2.49)^a^	8.14 (2.16)^b^	8.69 (2.22)^abc^
Male	10.02 (1.68)	10.05 (1.63)	9.87 (1.51)^ab^	9.64 (1.42)^abc^
Female	7.33 (1.02)***	7.47 (1.02)^a^***	7.54 (1.03)^ab^	7.52 (1.01)^a^***
Trunk muscle(kg)	21.94 (7.50)	21.91 (7.18)	21.20 (6.28)^ab^	23.35 (6.49)^abc^
Male	27.19 (4.17)	27.24 (3.96)	26.62 (3.65)^ab^	26.09 (3.34)^abc^
Female	19.49 (2.38)***	19.75 (2.41)^a^***	19.72 (2.36)^a^	19.70 (2.28)^***^
Visceral fat area	45.00 (42.00)	60.00 (51.00)^a^	72.00 (65.00)^ab^	101.00 (58.00)^abc^
Male	68.00 (24.75)	85.00 (36.00)^a^	113.00 (29.00)^ab^	117.00 (30.00)^abc^
Female	30.00 (18.00)***	39.00 (28.00)^a^***	50.00 (34.00)^ab^	59.00 (38.75)^abc^***
WHR	0.78 (0.11)	0.81 (0.09)^a^	0.83 (0.12)^ab^	0.89 (0.12)^abc^
Male	0.83 (0.06)	0.86 (0.06)^a^	0.92 (0.04)^ab^	0.94 (0.05)^abc^
Female	0.73 (0.08)***	0.78 (0.08)^a^***	0.81 (0.06)^ab^	0.82 (0.04)^abc^***

All data are presented as median (interquartile range)

*BMI* body mass index, *BF%* percent body fat, *WHR* waist-to-hip ratio

^***^p < 0.0001 versus male

^a^Significantly different from the 20–29-year group

^b^Significantly different from the 30–39-year group

^c^Significantly different from the 40–49-year group

### Associations of age and body composition

Linear regression analysis of body composition and age, as shown in Table 3, revealed that body composition indexes were highly significantly correlated with age in both men and women (*p* < 0.0001), where age was positively correlated with BMI, BF%, and total fat mass in both genders, and the correlations were greater in women (BMI: standard *β* = 0.24, BF%: standard *β* = 0.3, total fat mass: standard *β* = 0.23). Whereas age was negatively correlated with muscle mass in men (standard *β* =  − 0.14), it was positively correlated in women (standard *β* = 0.05). Age was significantly positively correlated with the visceral fat area and WHR in both men and women, and the correlation was greater in men (visceral fat area: standard *β* = 0.57, WHR: standard *β* = 0.64) than in women. It is noteworthy that age correlated with BMI, BF%, and muscle mass with smaller coefficients in both genders, while age correlated with fat mass (*R*^2^ = 0.54) in females and age correlated with visceral fat area (male: *R*^2^ = 0.32, female: *R*^2^ = 0.12) and WHR (male: *R*^2^ = 0.41, female: *R*^2^ = 0.21) in both genders with larger coefficients.

**Table 3:** Linear regression analysis between age and body composition indicators

Variables	Standard *β*	*t*	*p*	*R*^2^
BMI (kg/m^2^)				
Male	0.07	7.15	< 0.0001	0.005
Female	0.24	29.19	< 0.0001	0.06
BF%				
Male	0.16	16.34	< 0.0001	0.02
Female	0.3	37.7	< 0.0001	0.09
Total fat mass (kg)				
Male	0.06	5.94	< 0.0001	0.003
Female	0.23	28.49	< 0.0001	0.54
Total lean mass (kg)				
Male	− 0.14	− 14.83	< 0.0001	0.02
Female	0.05	5.86	< 0.0001	0.002
Visceral fat area (cm^2^)				
Male	0.57	71.3	< 0.0001	0.32
Female	0.35	44.04	< 0.0001	0.12
WHR				
Male	0.64	86.41	< 0.0001	0.41
Female	0.46	61.81	< 0.0001	0.21

### Obesity percentage under different criteria

**Fig. 7:**
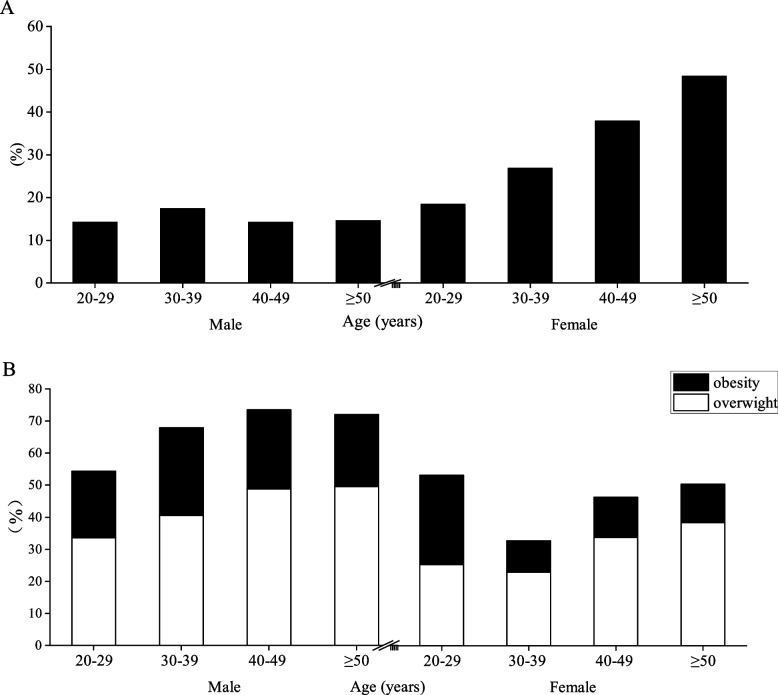
Column chart of obesity percentage under BF% (**A**) and BMI (**B**) criteria

**Fig. 8:**
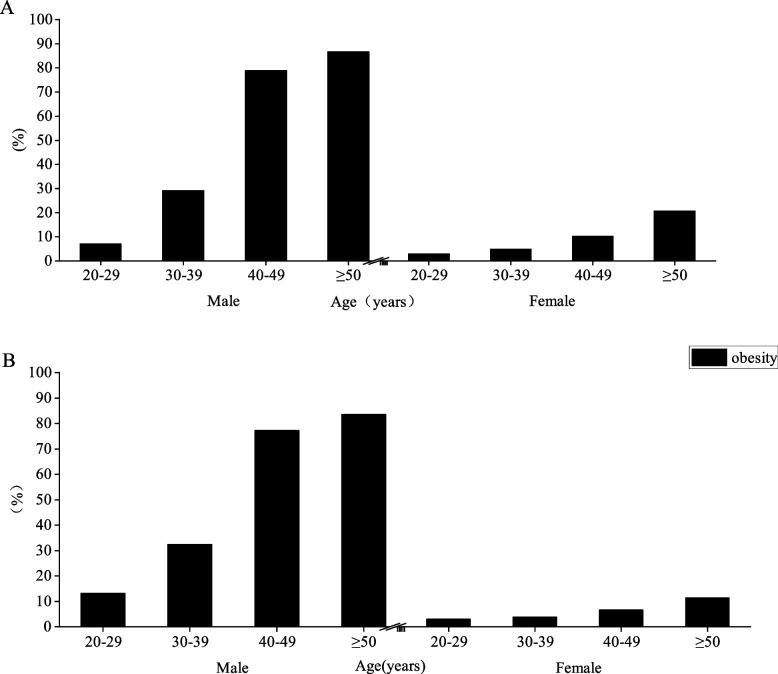
Column chart of obesity percentage under WHR (**A**) and visceral fat area criteria (**B**) criteria

Table 4 presents the percentages of all subjects classified as normal, overweight, and obese according to different body composition index criteria. Figures 7 and 8 show the percentages of overweight and obesity under different classification criteria. The results showed that the overweight rate of men under BMI criteria increased with age and exceeded 30% after 20–29 years of age, reaching 49.62% at ≥ 50 years of age, while the obesity rate of males increased and then decreased with age and reached a peak of 27.33% at 30–39 years of age, and the accumulation of overweight and obesity rates of males was found to reach a peak of 73.52% at 40–49 years of age. Compared with males, both overweight and obesity rates in females under BMI criteria appeared to decrease first and then increase, and both were smaller than in males. Under the BF% criterion, the obesity rate in men peaked at 17.41% at 30–39 years of age, while the obesity rate in women increased with age and the growth rate exceeded 10% in both 40–49-year and ≥ 50-year groups compared to the previous age group. Under the criteria of WHR and visceral fat area, the central obesity rate increased with age in both men and women, but it is noteworthy that the central obesity rate in men exceeded 80% at ≥ 50 years of age and increased sharply at 40–49 years of age, with both WHR and visceral fat area obesity rates increasing by 49.73% and 44.88% compared to 30–39 years of age. Although the central obesity rate in females also increased with age, the increase was smaller and the obesity rate did not exceed 30% in each age group.

**Table 4:** Percentage of obesity by BMI, BF%, WHR, and visceral fat area among subjects

Variables	BMI (%)			BF% (%)		WHR (%)		Visceral fat area (%)	
Age (years)	Normal	Overweight	Obesity	Normal	Obesity	Normal	Obesity	< 100 cm^2^	≥ 100 cm^2^
Male									
20–29	42.42	33.58	20.73	85.76	14.24	92.86	7.14	86.89	13.11
30–39	31.09	40.58	27.33	82.59	17.41	70.78	29.22	67.63	32.37
40–49	26.06	48.85	24.67	85.84	14.16	21.05	78.95	22.75	77.25
≥ 50	27.46	49.62	22.44	85.45	14.55	13.25	86.75	16.56	83.44
Female									
20–29	32.67	25.33	27.83	81.6	18.4	96.99	3.01	97	3
30–39	61.9	22.97	9.82	73.12	26.88	95.04	4.96	96.16	3.84
40–49	51.9	33.8	12.47	62.08	37.92	89.73	10.27	93.45	6.55
≥ 50	48.52	38.36	12	51.65	48.35	79.26	20.74	88.65	11.35

All data are presented as percentages (%)

*BMI* body mass index, *BF%* percent body fat, *WHR* waist-to-hip ratio

## Discussion

The present study covered adults aged 20–60 years in Beijing in a large age range with a large sample to show the age and gender characteristics of body composition and obesity distribution. The results of this study showed that (1) male BMI and fat mass peaked at age 40–49 years, whole-body and segmental muscle mass peaked at age 30–39 years, and BF%, WHR, and visceral fat area increased with age; (2) female BMI, fat mass, BF%, WHR, and visceral fat area increased significantly with age, and whole-body and segmental muscle mass changed with age; and (3) the Female obesity rate peaked at age 20–29 years under BMI criteria and the obesity rate increased significantly with age under BF%, WHR, and visceral fat area criteria.

### Age and gender characteristics of fat distribution

Age and gender are important influencing factors that affect body fat distribution. The results of this study showed that BMI and total fat mass peaked in Beijing adults aged 40–49 years, where BF% in men increased statistically with age but fluctuated less and never exceeded 25% and the visceral fat area was twice as high as in men and increased substantially with age. In females, BF%, total adiposity, and visceral fat area all increased significantly with age. Previously, Hong et al. [19] showed that BMI peaked in Korean male adults aged 40 years, BF% increased with age between 20 and 60 years, and both BMI and BF% increased with age in females. Tahara et al. [20] reported that both male and female BF% and adiposity peaked in Japanese adults aged 50–55 years. Although the total BF% in both genders tends to increase steadily with age, there are distinct gender-specific characteristics of fat distribution due to differences in physiological structure. Normal adult females have higher body fat than males, typically 25–35% in females with a BMI of 20–25 kg/m^2^ compared to 10–20% in males with the same BMI [21]. There are also significant gender differences in the distribution of body fat by region, with females typically having more peripheral subcutaneous fat tissue (SAT) (e.g. hips and femurs), whereas males tend to accumulate more central and visceral fat tissue (VAT) [22].

The molecular mechanisms associated with the gender characteristics of fat distribution are not known, but changes in body fat over the human lifetime correspond to changes in sex hormones, and age-related decreases in sex and steroid hormone levels lead to a shift from subcutaneous to central distribution of body fat in males and females [23]. Healthy females of reproductive age tend to store fat under the skin and in the lower body (forming a “pear shape”), and these subcutaneous adipose tissues are protective for females because they secrete higher levels of lipocalin and lower levels of pro-inflammatory adipokines [24]. In addition, subcutaneous adipocytes have a higher browning potential than visceral adipocytes, and brown fat consumes more energy and improves glucose homeostasis [24]. In contrast, after menopause, females experience a decrease in oestrogen and progesterone and an accumulation of abdominal fat (“apple shape”) [23]. A previous study showed that women gained about 5 kg of total fat and about 3 kg of trunk fat within 3 years after menopause [25]. Although male sex hormone concentrations do not change abruptly with age, testosterone concentrations in men decrease with age, which is associated with an increase in total body fat accumulation. In addition, increased fat leads to partial suppression of the hypothalamic–pituitary–gonadal axis in males, while substantial weight loss, such as after bariatric surgery, can return testosterone concentrations to normal [25]. Several previous studies have found that age-related hypogonadism in males is accompanied by an increase in visceral adiposity [26, 27]. The accumulation of adipose tissue in the abdominal fat depot exacerbates the risk of other diseases such as cardiovascular disease, type 2 diabetes, certain cancers, and metabolic syndrome [10, 11]. In summary, fat distribution between the sexes has significant age and gender characteristics, with young and middle-aged males more likely to store visceral fat and females more likely to store subcutaneous fat. With increasing age, both sexes are more likely to increase visceral fat accumulation.

### Age and gender characteristics of muscle distribution

Skeletal muscle mass is also an important indicator for evaluating adult health status, with relatively stable whole-body muscle mass from early adulthood to age 40 years and then beginning to decline naturally at a rate of approximately 1–2% per decade, also accompanied by a decline in strength, which in turn increases the risk of falls, frailty, and death [28, 29]. The results of this study showed that total muscle mass and muscle mass of body parts peaked at age 30–39 years and then decreased significantly among adults aged 20–60 years in Beijing, whereas total muscle mass in women fluctuated less and there was significantly greater muscle mass in the left and right upper and lower extremities in the 30–39-, 40–49-, and ≥ 50-year groups than in the 20–29-year group. Cui et al. [30] showed that muscle mass increased with age between 20 and 40 years and decreased after 50 years in Korean females. Karlsson et al. [31] reported that lean body mass decreased with age in Swedish women at 85 years. All of the above studies are somewhat inconsistent with the present study. There are many factors affecting the change in muscle mass; race, lifestyle, differential food intake, and exercise habits may be the main reasons. It has been shown that age-related declines in muscle strength typically occur at a faster rate than declines in muscle mass (i.e. approximately 2–3% per year) as a result of age-related deposition of musculature, innervation, and noncontractile material (fat and connective tissue) [32, 33].

Differences in body composition between the genders are evident from infancy, become most obvious after puberty (when boys experience accelerated growth bursts), and persist into old age [34]. In adulthood, at any given total body weight, young and middle-aged females have less muscle mass than males of the same age [35]. A recent study on gender dimorphism in skeletal muscle protein conversion noted that there were no significant gender differences in basal muscle protein synthesis and catabolic conversion rates between males and females aged 18–45 years and that hypertrophy in response to exercise training and atrophy in response to muscle deactivation were similar [36]. These findings agree with the results of the present study, and all of them indicate relatively stable muscle mass in both genders in young and middle age. With ageing, gender differences in muscle synthesis and catabolism begin to emerge between males and females, with older females having greater rates of muscle protein synthesis than males and less catabolism than males compared to matched older males (females: ~ 0.25%/year, males: ~ 0.40%/year) [37, 38]. However, it is noteworthy that the total muscle mass and left and right upper and lower extremity muscle content of women in this study were significantly greater in the 30–39-, 40–49-, and ≥ 50-year groups than in the 20–29-year group, which contradicts the results of previous studies. The results of previous studies have shown that age-related muscle loss in females coincides with the onset of menopause and that the rate of atrophy accelerates upon entering menopause [39]. Based on this result, the present study suggests that this may be related to the role of sex hormones in muscle metabolism. Although female sex hormones decrease abruptly during menopause, the main role of these oestrogens in middle adulthood is to maintain body fat and have an inhibitory effect on muscle protein synthesis, while menopause relieves this inhibition and increases the rate of muscle synthesis [40, 41]. In addition, residual confounding by other factors, such as long-term domestic work and dietary habits associated with the division of labour in the home, may also affect changes in muscle mass in females. In summary, muscle mass is consistently significantly higher in men than in women in adulthood, and changes in muscle mass are relatively stable with age in women, while muscle loss is more severe in men.

### Obesity distribution characteristics

Obesity is an important factor affecting cardiovascular disease. In China, obesity has become a major public health problem. According to the standards of the Chinese population, more than half of Chinese adults were overweight or obese in a recent national survey [42]. The results of this study showed that obesity rates under BMI criteria peaked in males aged 30–39 years (27.33%) and in females aged 20–29 years (27.83%) in Beijing. Under the BF% criterion, the obesity rate peaked at age 30–39 for males (17.41%) and increased significantly with age for females. The greater inconsistency of the obesity distribution results under these two criteria may be because BMI cannot distinguish between fat mass and lean mass, while males have more lean mass. However, it is important to note that the results of a recent survey on the distribution of obesity in a Chinese population of one million people found that the highest obesity rates were found in women aged 65–75 years and men aged 35–44 years under the BMI criterion [43]. In contrast, the results of this study showed that the highest obesity rates were found in men aged 30–39 years and women aged 20–29 years under the BMI criterion, suggesting a younger trend in the distribution of obesity among Beijing adults. A previous study investigating 22 developing countries with young adults from 22 universities with an average age of 20.8 years found overweight and obesity rates of 22%, with 24.7% for males and 19.3% for females [44]. In contrast, the obesity rate in the United States in 2017–1018 was 40.03% for males and 39.7% for females aged 20–39 years [45]. This is indicative of obesity in young adults, and this study suggests that adults aged 20–29 years are vulnerable to social and environmental factors such as financial independence, high-calorie “ready-to-eat” foods, sedentary lifestyles, and lack of physical activity that make them more likely to be obese. Central obesity is more strongly associated with total mortality and cardiovascular mortality than overall obesity [10, 11].

The results of the present study showed that central obesity rates were greater in males than in females in each age group under WHR and visceral fat area criteria and that central obesity rates increased with age in both males and females. A previous study with a large sample of 15,184 US adults with a mean age of 45 years found that 70.2% of adults met the WHR criteria for central obesity [11]. In contrast, the results of this study showed low rates of central obesity in males and females at 20–39 years of age, while the rate of central obesity in males increased significantly after 40 years of age, reaching more than 70% in both cases. A recent epidemiological study found that in the past 20 years, overweight and obesity were clustered in adults in North China, Northeast China, and the Bohai Rim, with the highest prevalence of overweight and obesity in Beijing [42]. This may be the result of a combination of lifestyle, physical fitness, economic development, social welfare, and cultural background. Therefore, it is important to encourage both the prevention of central obesity with age and the rejuvenation of obesity by encouraging more physical activity in younger populations and actively reversing the diseases associated with obesity.

## Conclusion

In summary, the results of this study suggest that changes in body composition associated with Beijing adults aged 20–60 years vary by gender across the age range. With age, males had greater potential for stored visceral fat, greater loss of muscle mass, and higher rates of central obesity. In contrast, females showed significant increases in BF% with age, stable muscle mass, and steady increases in both overall and central obesity rates. Therefore, a better understanding of the characteristics of body composition and obesity distribution in Beijing adults should be developed to actively prevent the occurrence of obesity.

### Supplementary Information

**Additional file 1: Table S1.** Statistical table of occupational classification of different age groups.

## Data Availability

All data generated or analysed during this study are included in this published article.

## Abbreviations

BMI: Body Mass Index
BF%: Percentage of body fat
BIA: Bioelectrical impedance analysis
CT: Computed tomography
DXA: Dual-energy X-ray absorptiometry
IQR: Interquartile range
LMS: Least mean square
SAT: Subcutaneous fat tissue
VAT: Visceral fat tissue
WHR: Waist-hip ratio
