# Supplementary material for: Age- and sex-related trends in body composition among Beijing adults aged 20–60 years: a cross-sectional study
Source: BMC Public Health. 2023 Aug 10;23:1519. doi: 10.1186/s12889-023-16459-0 (PMC10413732; doi:10.1186/s12889-023-16459-0)
Supplement: Supplementary file 1 — Additional file 1: Table S1. Statistical table of occupational classification of different age groups. [file 12889_2023_16459_MOESM1_ESM.docx]

Table S1 Statistical table of occupational classification of different age groups

|  | Age category (years) | | | |
| --- | --- | --- | --- | --- |
| Characteristics | 20-29 (n=5253) | 30-39 (n=8325) | 40-49 (n=7054) | 50-60 (n=3782) |
| Occupation Type(%) |  |  |  |  |
| Services | 1224(23.3%) | 1990(23.90%) | 1601(22.70%) | 831(21.97%) |
| Industry | 517(9.84%) | 698(8.38%) | 580(8.22%) | 330(8.73%) |
| Education | 575(10.95) | 623(7.48%) | 858(12.16%) | 462(12.22%) |
| Architecture | 432(8.22%) | 589(7.08%) | 576(8.17%) | 244(6.45%) |
| Transportation | 459(8.74%) | 769(9.24%) | 512(7.26%) | 372(9.84%) |
| Finance | 308(5.86%) | 409(4.91%) | 405(5.74%) | 225(5.95%) |
| Civil Service | 643(12.24%) | 1395(16.76) | 889(12.60%) | 534(14.12%) |
| Others | 1108(21.09%) | 1852(21.92%) | 1633(23.15%) | 722(19.09%) |
